# A New Vision for the Journal of Child and Adolescent Trauma

**DOI:** 10.1007/s40653-021-00347-z

**Published:** 2021-02-17

**Authors:** Dominic McSherry

**Affiliations:** School of Psychology, Ulster University, Cromore Road, Coleraine, Northern Ireland

## Introduction

Hello everyone! My name is Dominic McSherry and I am the new Editor in Chief for the Journal of Child and Adolescent Trauma (JCAT). I’ve taken over from Bob Geffner, who led the journal from its inception 15 years ago. Given that this Special Issue reflects Bob’s final contribution, I’d like to take this opportunity to acknowledge his longstanding commitment to addressing child and adolescent trauma, and to wish him well for the future. As a reflection of this contribution, he will be included as founding Editor in Chief on the journal website.

My position as Editor in Chief officially commenced on the 1st January 2021. However, I was appointed in August 2020, and took over management of all submissions to the Journal from the 1st September 2020. This early de-facto four-month leadership position was very useful in two ways. First, it enabled me to familiarise myself with the Editorial Management system and the many Editorial Board members and reviewers who were supporting the work of the journal. Second, it provided a reasonable time period for me to plan the restructuring of the journal in terms of a new editorial management team and updated Board, and to begin to refine the aims and scope of the journal in line with my new vision.

## My Background

There is a short biography of me on the journal website for those interested, but in summary I am a developmental psychologist at Ulster University in Northern Ireland, working in both the School of Psychology and our Faculty-level multidisciplinary Institute of Mental Health Sciences. For the last 20 years my research has been focused on early childhood experience of early adversity and trauma, primarily as a result of maltreatment, subsequent entry to the care system, and for some, being adopted from care.

### My Beliefs about Childhood Trauma

Trauma has shadowed humankind since our earliest days. As our ancestors struggled to survive the harshest of conditions, our physiological systems evolved to protect us from the vast array of mortal perils that we faced. Many of these threats were environmental, such as famine, drought, extremes of temperature, flooding, fire, and earthquake. But they were also human-made, such as intra and inter-group/familial conflict and maltreatment. There was also the blunt reality of the fragility of life, brutally reflected for example by the sudden death of a loved one. Yet, whether environmental, self-inflicted, or human frailty, the outcome of these struggles remained consistent, physical and emotional pain and suffering.

This is as true today as it was 10,000 years ago. Children continue to suffer from environmental threats, the fragility of life, and the awful legacy of conflict and maltreatment. We protect ourselves from these threats and stresses using the same basic internal physiological guard-system that protected our early ancestors from imminent danger, the adrenal response, the compulsion for fight, flight or freezing. Then, as now, the primary purpose was survival. Then as now the primacy of the survival response can have detrimental psychological effects, particularly where stress responses are prolonged, and the environment in which they occur is frightening, unsupportive and unpredictable. Does this mean that I believe we should simply accept child trauma as an inevitable part of the human condition? The answer is ‘Yes’ and ‘No’.

‘Yes’, because our planet continues to threaten human survival through famine, flooding, extremes of weather and temperature, earthquakes, tsunami, and of course as we know only too well from recent experience, viral infection. We can and should try to influence the likelihood of such events, for example in relation to climate change, attempting to control for greenhouse gas emissions, reduce carbon emissions, protect the ozone layer, develop international treaties, such as the Paris Agreement, to reduce global warming. But, no matter how we try to manage these processes, disasters will still occur, and children will continue to be traumatised as a result. In these circumstances, we should explore what is the best way to respond to such natural disasters. This is an area of work where I’m hoping to see a greater influx of papers into JCAT as we move forward. A renewed focus on natural disaster and evidence of what works best for children in terms of firstly preventing trauma, and secondly, where this has occurred, treating it effectively.

‘No’, because there are multiple levels of trauma for children that are the direct consequence of human action or inaction. The poverty, social deprivation and inequality that continues to tarnish human societies locally and globally, is a key driver of the war, conflict and maltreatment that leads to the trauma that too many children and adolescents continue to experience. We have the capacity at local and global levels to significantly reduce these human-made drivers of child trauma. What we lack is enough collective desire and conviction to do so. Moving forward then, I would like to see further evidence of the impact of these traumas on children, and how these can be experienced into adulthood, and what works in reducing incidence and treating symptoms. However, I would be very keen to see a focus on some of the larger structural issues that can be the driver of these difficulties, and theoretical, conceptual and practical suggestions as to how these social deficits can be alleviated.

### My Vision for the Journal

Building upon the Journal’s already established strong foundations, my vision is to create a structure that enables us to more fully understand and address child and adolescent trauma. As such, there are a number of key areas that I plan to focus on for further development within the journal. These are: Internationalisation; a biopsychosocial approach; multidisciplinarity; interprofessionality; and moving beyond child maltreatment*.*

#### Internationalisation

A key aspect of addressing some of the challenges I’ve just raised is ensuring that the journal is of relevance to an international audience. A central tenant of my beliefs about child trauma is the universality of human experience. We all share the same human body that has evolved to respond to threat via our survival mechanisms. Of course, the environment that each of us experiences can differ dramatically. But, whatever the environment or cultural context, it is the same human being, with the same physiological response system, trying to survive the world one day at a time. Consequently, I believe that there is much scope for us to learn from each other across the globe about our experiences of trauma and approaches that have been developed, historically or more recently, to prevent and address child and adolescent traumatic symptomatology.

I’ve already taken a number of steps to try and achieve that goal. First, to establish a new associate editorial team for the journal that is reflective of expertise at an international level. Second, to further open up membership of the Editorial Board to expertise at a global level. The new Associate Editorial team consists of members from the US (David Brodzinsky; Arne Graff; Patricia Kerig; Bruce Perry; and Gina Samuels), Northern Ireland (Gerry Leavey; Mark Shevlin; Tom Teggart; and Mark Tully), England (Muthanna Samara; Karen Treisman; and Panos Vostanis), Republic of Ireland (Robbie Gilligan), India (Avinash Desousa), Italy (Chiara Ionio), Canada (Nadine Lanctôt), and South Africa (Adrian Van Breda). Additionally, Muthanna, a UK-based academic, is originally from the Middle East, so he is able to bring an important perspective from a sadly troubled region of the world. Hopefully this widening of the geographic spread of the journal leadership will be reflected in a greater geographically diverse spread of submissions.

#### Biopsychosocial Approach

I firmly believe that we cannot fully understand child trauma without applying a lens that is able to capture meaning and interaction at biological, psychological and societal levels. Some would refer to this as gene-environment interaction. However, I’m somewhat uncomfortable with that terminology as to some degree it overlooks intermediate processes, the psychology of the person, their thoughts, feelings and actions, which we know are critical to our understanding of trauma. That said, moving forward I would like to see more of a focus within submissions on firstly human biology and the role that our genetic make-up can have in terms of our susceptibility or resilience to the initial processing of traumatic events and the management of any stress response that may or may not occur. Secondly, I would like to see more contributions that address systemic factors that can provide a rich antecedent environment for child and adolescent trauma, such as poverty, social deprivations, and inequality and discrimination, either on the basis of race, ethnicity, gender, sexual orientation, or class. In my view, a complex circular interplay of biological, psychological and social factors will be involved when considering all human responses to trauma, be they minimal or of significant harm. This will certainly be the case when considering child and adolescent trauma.

#### Multidisciplinarity

Given my focus on a biopsychosocial approach, it not surprising then that I believe our understanding of child and adolescent trauma is best advanced when academics and scholars in the biological, psychological and social fields work together to develop novel and advanced understanding, and preventative and treatment approaches. This has been a core dimension of my own research over the last 20 years, and I’ve seen at first hand the rewards it can bring. As such, it remains embedded within the work that I do with my psychology colleagues and also the diverse range of colleagues I work with across the Faculty in our Institute of Mental Health Sciences at Ulster University. The journal already had a wide range of disciplines specified within the aims and scope on the webpage, and that has now been enhanced by adding in education and nursing, two disciplines that are very often at the coal face of societies’ responses to child trauma. Consequently, we have much to learn from and share with these colleagues within this journal.

#### Interprofessionality

There has been no stronger evidence of the need for interprofessional cooperation than what we’ve experienced across the globe from the COVID-19 pandemic. Although often used in a flippant way, the term that ‘we are all in this together’ has never been more apt. In my view, there are multiple levels of knowledge and understanding of child and adolescent trauma embedded across all our communities, be that on a university campus, a social service department, a hospital ward, a science laboratory, or in a school. I want this journal to be a conduit for the free expression and sharing of that knowledge and understanding, in a way that democratises the source, and privileges no particular perspective. This has been reflected in the make-up of the new Associate Editorial team, with seven academic/practitioners, six academics, and four practitioners included, and the Editorial Board, which reflects a rich diversity of academic and practitioner backgrounds. Furthermore, the capacity for academic research to have impact at the local and global level is greatly enhanced if it is generated by and shared with those professionals who deal with the reality of child and adolescent trauma, in childhood and adulthood, on a day and daily basis.

#### Beyond child maltreatment

Although my own research over the last 20 years has been focused on trauma related to the early experience of child maltreatment, I do not wish the journal to become an echo chamber for that singular perspective. Since taking over as de facto Editor in Chief back in September 2020, it has become clear that is certainly the dominant consideration within the journal currently, despite the fact that the aims and objectives specify a wide range of area of interest, including: loss, natural disasters, and political conflict; exposure to or victimisation from family or community violence; physical injury, disease, and painful or debilitating medical treatments; racial, ethnic, gender, sexual orientation (just included) or class discrimination. Two further key areas of interest have also been added since my tenure as EiC commenced: poverty, social deprivation, and inequality; and barriers and facilitators on pathways to recovery.

To fully understand and address child and adolescent trauma we need to understand and address it in its totality, across all contexts, to collectively explore and learn from the variation and similarities in experience, and the multiplicity of perspectives. Over recent years societies across the world have been ravaged by ethno-political wars and conflicts, major devastation has been caused to our living environment through natural disasters involving drought, flooding, and fire, and we have been facing perhaps the biggest global health crisis of the century with the COVID-19 pandemic. Of course in all of this the impact of child maltreatment will remain a key concern, but so will all of the other contexts of child trauma, and I hope my tenure as EiC will see a major growth in research and scholarship across this broad range of critical domains.

## Conclusion

I am deeply honoured to have been tasked with the role of leading the Journal of Child and Adolescent Trauma over the next five years, and in the calibre of academics, scholars and practitioners who have so graciously committed to support this journey, both on the Associate Editorial Team, and the Editorial Board. I have set out a general vision of how I would like to see the journal develop, and this has been forged through very helpful discussions with many colleagues, including Associate Editors and Board member. When we look around us today we see clearly that the world faces many challenges, none perhaps more significant than how we deal with the COVID-19 pandemic and respond to the avalanche of trauma that is likely to follow in its wake, particularly for children and adolescents. But, Science and the Arts have shone a light on the resilience of the human spirit in the face of such significant adversity, and we are part of that spirit, the spirit of freedom, knowledge, enlightenment, and striving to find a better path for our fellow human beings, especially our children and young people. This journal is an opportunity to make a positive difference. To better understand the biopsychosocial dimensions of child and adolescent trauma, and to provide policy makers and practitioners with evidence of what works best in terms of prevention and treatment. Let’s continue to collectively make this happen in the Journal of Child and Adolescent Trauma (JCAT).

